# Right Coronary Artery to Left Ventricular Fistula Complicated by Symptomatic Arrhythmia

**DOI:** 10.7759/cureus.62217

**Published:** 2024-06-12

**Authors:** Atif AlQubbany, Yazeed Alqurashi, Alaa Meer, Abdulbari Aboud, Amin Zagzoog, Ahmed Krimly

**Affiliations:** 1 Cardiology, Ministry of National Guard Health Affairs, King Abdullah International Medical Research Center, Jeddah, SAU; 2 Adult Cardiology, King Faisal Cardiac Center, King Abdulaziz Medical City, Ministry of National Guard Health Affairs, Jeddah, SAU; 3 Cardiology, King Faisal Cardiac Center, King Abdulaziz Medical City, Ministry of National Guard Health Affairs, Jeddah, SAU; 4 Medicine, Ministry of National Guard Health Affairs, Jeddah, SAU; 5 Medical Research, King Abdullah International Medical Research Center, Jeddah, SAU; 6 Medical Research, King Saud Bin Abdulaziz University for Health Sciences, Jeddah, SAU

**Keywords:** transcatheter closure, cardiovascular intervention, cardiac electrophysiology, coronary arterial fistula, ventricular arrhythmia, adult congenital heart disease (achd)

## Abstract

Coronary cameral fistulas (CCFs) are rare and are characterized by an abnormal connection between a coronary artery and any of the four chambers of the heart. Most cases of CCFs are asymptomatic. The most common presentation in symptomatic patients includes chest pain or heart failure; however, arrhythmias are rarely associated. We report the case of a 32-year-old male previously unknown to have any medical illnesses. He presented to the clinic with complaints of frequent palpitations, necessitating recurrent admissions. His electrocardiograms revealed regular wide complex tachycardia with a right bundle branch block pattern, suggestive of fascicular ventricular tachycardia. During hospitalization, an elective coronary angiography showed a large CCF originating from the right posterior descending coronary artery and draining into the left ventricle. Moreover, cardiac magnetic resonance imaging did not show any scar or evidence of cardiomyopathies. The patient underwent a successful catheter-based right coronary artery to left ventricular fistula occlusion with coils. In addition, the patient underwent a complex electrophysiological study with three-dimensional mapping and ablation. The presented case underscores the rarity and complexity of such clinical presentations. It also highlights the importance of a multidisciplinary approach in addressing this unique cardiac anomaly.

## Introduction

Coronary cameral fistulas (CCFs) are rare and characterized by an abnormal connection between a coronary artery and any of the four chambers of the heart [1]. The prevalence of coronary artery fistulas (CAFs) was initially thought to be 0.05-0.25% of the population. However, coronary computed tomography angiography (CTCA) has been found to detect more CAFs, with a prevalence of 0.9% [2]. Based on the type of communication with the cardiac chamber, they are classified as arterio-luminal (direct communication with the cardiac chamber) or arterio-sinusoidal (communication via the sinusoidal network rather than direct communication) [3]. CCFs drain into the right-sided chamber in approximately 90% of cases [4]. Although the most common fistulous connection occurs between the right coronary artery (RCA) and right ventricle, it can also originate from the left coronary system and drain into the left heart chambers.

Most cases of CCF are asymptomatic. The most common presentation in symptomatic patients includes chest pain or heart failure; however, arrhythmias are rarely associated [5]. Here, we report a case of large CCF originating from the right posterior descending coronary artery (RPDA) and draining into the left ventricle (LV) that presented with recurrent fascicular ventricular tachycardia (VT).

## Case presentation

A 32-year-old male previously unknown to have any medical illness presented to the clinic with complaints of frequent palpitations associated with dyspnea, diaphoresis, and nausea, necessitating recurrent admissions. The patient denied taking any medication, illicit drugs, or consuming alcohol. One of his previous electrocardiograms (ECGs) revealed regular wide complex tachycardia with a right bundle branch block pattern (RBBB), suggestive of fascicular VT. Additionally, transthoracic echocardiography (TTE) showed suspicion of a ventricular septal defect (VSD), presumed to be a possible cause of his fascicular VT.

**Figure 1:**
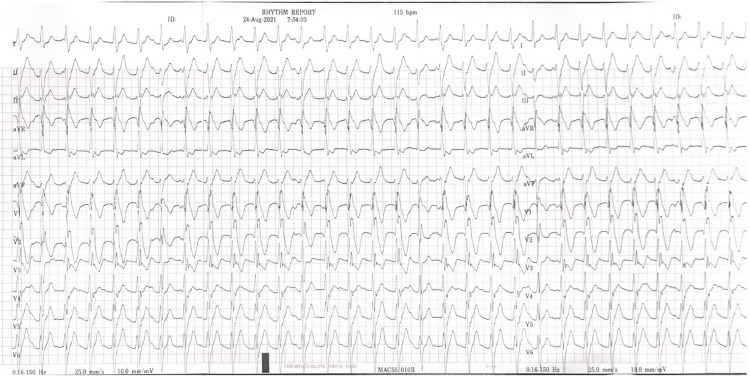
Regular wide complex tachycardia with right bundle branch block pattern, suggestive of fascicular ventricular tachycardia.

Upon initial evaluation, he exhibited normal sinus rhythm on the initial ECG. Holter monitoring for 72 hours showed no documentation of any arrhythmia, and he had no symptoms during the observation period. Furthermore, his TTE at our hospital showed evidence of abnormal color Doppler flow, suggestive of VSD (Video 1). During one episode of palpitations and lightheadedness, the patient presented to our emergency department, where the ECG revealed fascicular VT (Figure 1).

**Video 1:** Abnormal color Doppler suggestive of ventricular septal defect.

**Figure 2:**
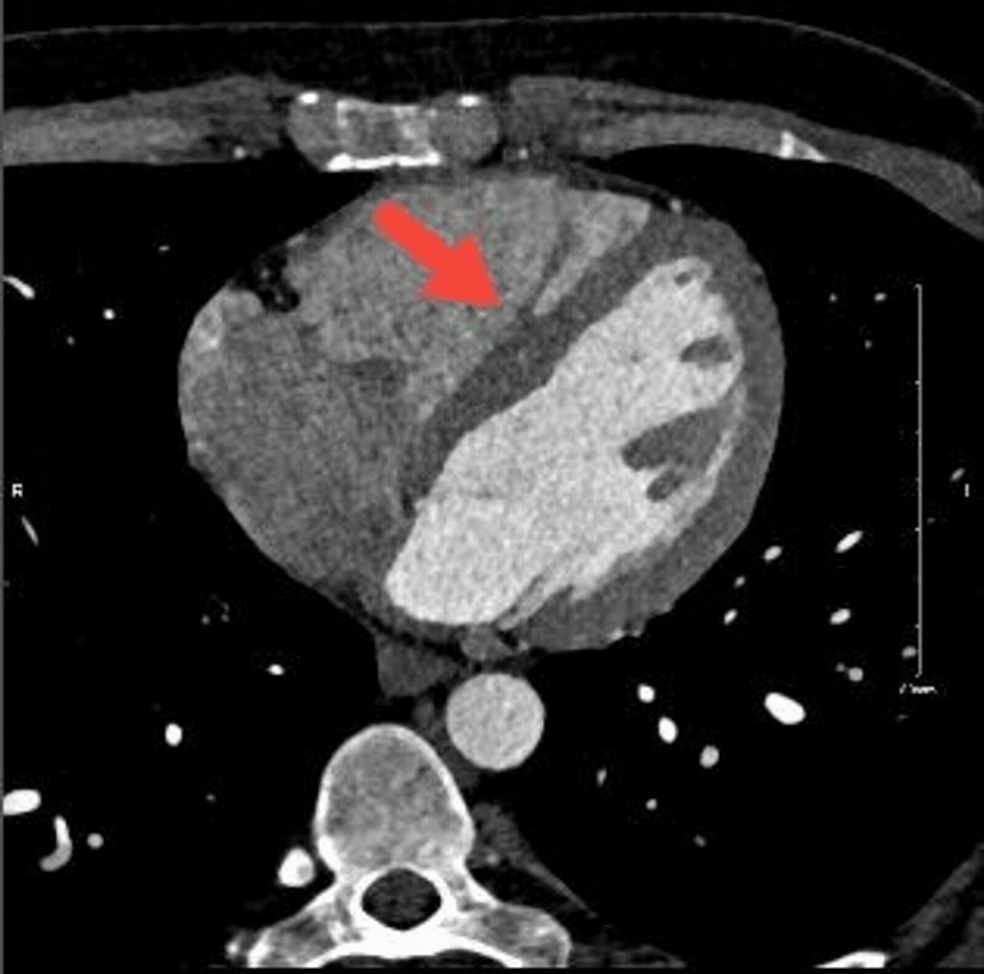
Coronary computed tomography angiography showing the posterior descending artery terminating with a large fistula (arrow) communicating with the left ventricle.

An elective coronary angiography was performed, revealing patent epicardial coronary arteries but showing an anomalous large connection between the posterior descending artery (PDA) and the LV. Additionally, a small fistula between the distal obtuse margin (OM) and the LV was observed (Video 2). To further define the origin and course of the anomalous coronary arteries, CTCA was performed. CTCA demonstrated an anomalous artery originating from the right coronary sinus, sharing the same ostium with the RCA, coursing trans-septally, and bifurcating into two branches within the septum. One of those branches coursed through the septum to the posterior interventricular groove to form the PDA. This PDA finally terminated with a large fistula measuring 5 mm, which communicated with the LV (Figure 2).

**Video 2:** Anomalous large connection between the posterior descending artery and the left ventricle.

**Figure 3:**
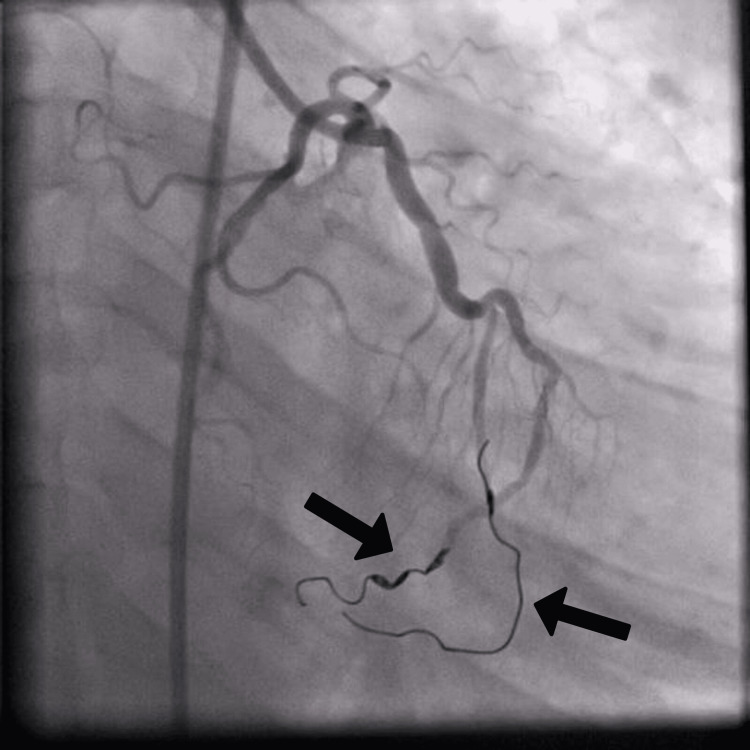
Transcatheter closure of the fistula using coils (arrows).

Cardiac magnetic resonance imaging was performed to investigate whether there was any myocardial scar explaining this recurrent VT. It did not show any scar or evidence of cardiomyopathies. The patient underwent a successful catheter-based RCA to LV fistula occlusion with coils (Figure 3). Moreover, the patient underwent a complex electrophysiological study with three-dimensional mapping and ablation. He recovered and remained asymptomatic for two years.

## Discussion

CAFs are a rare condition that can be congenital or, less commonly, acquired due to prior intervention or heart disease. CAFs are usually found incidentally during a coronary angiogram (CAG). Fistulae vary in their communicating structures between the coronaries and surrounding vessels. In our case, the fistulae were between the coronaries and heart chambers. In such rare cases, they are known as CCFs, which are found in 0.1-0.2% of CAG cases [6].

Previous case series have concluded that most CCFs drain into the right ventricle (90% of cases), producing a left-to-right shunt that can cause overloading of the pulmonary vasculature. Such patients may have a more symptomatic presentation. In contrast, only 3% terminate in the LV, as in our case. Thus, the presentation differed in this case, with CCF manifesting as arrhythmia or ischemic chest pain due to a phenomenon known as “Steal.” When the LV is in the diastolic phase, the gradient pressure forces blood to flow to the LV and neglect the distal territories to the CCF [7].

According to Stojišić-Milosavljević et al., dual CAFs, as in our case, are very rare and estimated to occur in only 5% of patients with this anomaly. The most common site of fistula termination is at the pulmonary arteries (>50%) [8].

Furthermore, fistulae can be categorized by size concerning the largest coronary artery that does not feed the fistula. For example, small fistulae are less than one times the diameter of the largest coronary artery not feeding the fistula, medium fistulae are one to two times the diameter, and large fistulae are greater than two times the diameter. Small fistulae usually do not require intervention and sometimes close without intervention [9].

Regarding management, there are no current specific guidelines, and the latest recommendation from the American College of Cardiology/American Heart Association in 2018 advised using a heart team approach to decide about possible intervention, either medically, surgically, or by transcatheter closure techniques [9].

Sager et al. reported the case of a 58-year-old female who was incidentally found to have multiple large fistulae causing a network connection from the left circumflex coronary artery to the LV. No intervention was done given the patient’s age, preserved LV function, and asymptomatic status [10].

On the other hand, a case of a middle-aged male who presented with palpitations and was found to have an RCA-LV fistula with RCA diffuse ectasia was treated differently. He was managed by surgical fistula repair, resection/reconstruction of the RCA, and coronary artery bypass grafting, and he had a favorable clinical outcome after one year of follow-up [11].

However, the surgical approach is not always successful. Wang et al. published the case of a 33-year-old male who was investigated for chest pain and found to have an RCA-LV fistula. He underwent surgical repair, but on the third day after the operation, an echocardiogram showed that the fistula had recurred. The patient was discharged on day seven and later developed a COVID-19 infection. He presented with palpitations and chest pain, which were eventually managed with metoprolol [12].

Furthermore, transcatheter closure is increasingly used in CAFs. Jiang et al. reported an excellent outcome in a middle-aged woman who presented with chest pain and congestive heart failure symptoms that resolved after RCA-LV fistula closure using a coil [13].

Furthermore, our literature search for patients with CAFs who presented with VT revealed multiple case reports with different management approaches and outcomes. For example, Hickman et al. reported the case of a 40-year-old male who presented with syncope while jogging. In the emergency room, he was found to have atrial fibrillation, followed by VT and ventricular fibrillation, which were resuscitated by DC shock. Later, he was found to have a fistula from the circumflex artery to the coronary sinus. As he had another VT episode during a stress test, surgical ligation was done. In the five-year follow-up, the patient remained asymptomatic [14].

Another case by Hu et al. reported a left anterior descending artery to right ventricle fistula in a 38-year-old male referred to as a case of recurrent VT. The patient recovered after ablation of the lesion in the middle cardiac vein and was followed for six months without arrhythmia [15].

In addition, Stojišić-Milosavljević et al. described the case of a 23-year-old male who presented with recurrent VT and was found to have two CAFs to the pulmonary artery originating from the RCA, which were managed by surgical ligation. The other CAF was from the left anterior descending artery and via a network of thin vessels, and no intervention was done for it. After the ligation, the patient continued to have recurrent VT. As a result, he underwent three electrophysiology studies without provoking VT and was eventually managed by an implantable cardioverter defibrillator. During the first year of follow-up, four episodes of VT were terminated [8].

## Conclusions

The presented case of fascicular VT associated with a large fistula between the RCA and the LV underscores the rarity and complexity of such clinical presentations. The successful management involving catheter-based coil closure of the fistula, coupled with cardiac ablation, highlights the importance of a multidisciplinary approach in addressing this unique cardiac anomaly. This case adds to the limited body of evidence regarding the optimal management of such cases, emphasizing the need for further research and comprehensive studies to establish standardized protocols.
